# Functional Characterization of OsWRKY7, a Novel WRKY Transcription Factor in Rice

**DOI:** 10.3390/life15121852

**Published:** 2025-12-02

**Authors:** Yuting Wei, Zhengyu Si, Haozhe Zhang, Can Hu, Bo Liu, Chenfan Zheng, Yuanyuan Tan, Qingyao Shu, Meng Jiang

**Affiliations:** 1 Hainan Institute, Zhejiang University, Yazhou Bay Science and Technology City, Sanya 572025, China; wytt@zju.edu.cn (Y.W.); sizhengyu@zju.edu.cn (Z.S.); 22316167@zju.edu.cn (C.H.); 22316131@zju.edu.cn (B.L.); 22216155@zju.edu.cn (C.Z.); tanyy@zju.edu.cn (Y.T.); qyshu@zju.edu.cn (Q.S.); 2 National Key Laboratory of Rice Breeding and Biology, The Advanced Seed Institute, Zhejiang University, Hangzhou 310058, China; 3 School of Chemistry, Faculty of Science, University of Bristol, Bristol BS8 1TS, UK; zhz2846673392@126.com

**Keywords:** rice, WRKY, transcription factor, expression characteristics

## Abstract

Transcription factors (TFs) orchestrate plant growth and development, yet the functional landscape of many TF gene families remains incomplete. Here, we systematically characterize *OsWRKY7*, an unannotated WRKY TF in rice. Phylogenomic analyses revealed that the *WRKY7* subfamily originated in basal angiosperms and evolved under strong purifying selection. We demonstrate OsWRKY7 functions as a WRKY transcriptional activator, with its activity uniquely encoded within the N-terminal domain—a distinctive mechanism among WRKY proteins. The promoter is enriched with cis-elements responsive to hormone and stress signaling, and the gene shows predominant expression in seeds. Strikingly, haplotype analysis revealed exceptionally low genetic diversity at the *OsWRKY7* locus, suggesting evolutionary constraint or a recent selective sweep. Our findings establish *OsWRKY7* as a conserved regulator with unique molecular features, specifically the WRKY domain, providing a strategic target for both fundamental research and crop improvement.

## 1. Introduction

*Oryza sativa* L. (rice) is a staple food for more than half of the global population and stands as a core crop for safeguarding food security [1,2,3]. The adaptive responses of rice to abiotic and biotic challenges, as well as its growth and development, are directed by complex gene regulatory networks [4,5,6]. As key nodes within this network, transcription factors (TFs) maintain rice’s adaptability to environmental signals by binding to cis-elements in target genes and regulating their expression [7,8,9]. Among the plant-specific transcription factor families, the WRKY family has emerged as a core regulator of rice’s stress tolerance, disease resistance, and growth and development [10]. The WRKY family, a class of plant-specific transcription factors, constitutes a central regulatory module that coordinates rice stress tolerance, immunity, and agronomic traits [4,11]. WRKY proteins are characterized by their namesake WRKY domain, a 60-residue region that contains the invariant WRKYGQK heptapeptide at its N-terminus and a C_2_H_2_-type zinc-finger motif [12]. While WRKYGQK is the canonical sequence, variants such as WRKYGKK exist, with the consensus broadly defined as W(R/K)(K/R)Y [13,14]. This domain confers the ability to bind W-box cis-elements, thereby modulating transcriptional outputs [4,10]. Given their profound influence on critical phenotypes, members of the rice WRKY family represent high-value targets for molecular breeding strategies designed to engineer stress-resilient and high-yielding varieties [15].

WRKY transcription factors are hierarchically classified into three principal groups (I, II, and III) based on two structural features: the number of WRKY domains (two in Group I, one in Groups II and III) and the type of zinc-finger motif (C_2_H_2_ for Groups I and II, C_2_HC for Group III) [11]. This classification provides a structural framework for understanding the functional diversity of this key protein family [11,15,16]. Members of Group I often exhibit specialized roles in stress adaptation [17]. For instance, *OsWRKY53* acts as a negative regulator under both cold and salt stress [18,19]. It attenuates cold tolerance by repressing gibberellic acid biosynthesis, thereby compromising pollen fertility [19], and impairs salt tolerance by directly suppressing the expression of key salt-tolerance genes such as *OsMKK10.2* and *OsHKT1;5* [18]. Group II is the largest and most functionally diverse [17]. Its members frequently exert opposing effects within the same stress context. Under cold stress, *OsWRKY71* [20] and *OsWRKY76* [21] act as positive regulators, whereas OsWRKY63 functions as a negative regulator by suppressing *OsWRKY76* expression [22]. Similarly, in drought responses, *OsWRKY11* enhances tolerance [23], while *OsWRKY5*, *OsWRKY55*, and *OsWRKY114* negatively impact it [24,25,26]. During salt stress, however, Group II members such as *OsWRKY54* predominantly serve positive roles—for example, by activating *OsHKT1* to maintain Na^+^/K^+^ homeostasis [27]. In Group III, *OsWRKY70* and *OsWRKY94* promote cold tolerance by upregulating cold-responsive genes such as *OsLti6b* and *OsICE1*, while fine-tuning the balance between defense and growth [28]. In contrast, *OsWRKY10* acts as a negative regulator of thermotolerance; its inhibitory effect is alleviated through interaction with *VQ8*, thereby enhancing heat stress resilience [29]. Collectively, WRKY proteins from different groups constitute a sophisticated and interconnected regulatory network that enables rice to coordinate adaptive responses to diverse environmental challenges [4,30].

While the WRKY transcription factor family is well-established as a central regulator of crop stress responses, the functional characterization of *OsWRKY7* remains incomplete. Based on the nucleotide and amino acid sequence analysis of the poorly studied OsWRKY7, we hypothesize that this protein plays a role in stress adaptation due to the presence of conserved WRKY motifs and nuclear localization signals, but it also exhibits unique characteristics, such as distinct C-terminal domain organization. To investigate this hypothesis, we will systematically examine its evolutionary lineage, protein architecture, subcellular localization, and transcriptional activation potential. Furthermore, we will profile its expression patterns under various abiotic stresses and validate its binding to key cis-regulatory elements. Through this comprehensive approach, our work aims to elucidate the molecular mechanisms underlying *OsWRKY7* function in environmental adaptation, ultimately establishing its value as a candidate gene for developing stress-resilient rice varieties.

## 2. Materials and Methods

### 2.1. Cloning and Sequence Analysis of OsWRKY7

Protein sequences of *WRKY7* homologs from diverse plant species were retrieved from the NCBI database. Phylogenetic analysis was performed with MEGA 7.0 [31] to reconstruct the evolutionary relationships among these sequences, and a phylogenetic tree was generated. The tree was then visualized and refined using the Interactive Tree of Life (iTOL) (available online: https://itol.embl.de/ (accessed on 28 November 2025) (iTOL) [32]).

To investigate closely related lineages, we selected homologous protein sequences from plant species phylogenetically adjacent to *OsWRKY7.* Multiple sequence alignments (MSAs) of these homologs—including *OsWRKY7* and its orthologs from other plants—were generated using DNAMAN (version 9) [33] software. Homology models of the target proteins from representative species were subsequently constructed using the SWISS-MODEL (available online: https://swissmodel.expasy.org/ (accessed on 28 November 2025)) [34].

### 2.2. Bioinformatic Analysis of OsWRKY7

For comprehensive protein characterization, we utilized several tools. We used ProtScale (available online: https://web.expasy.org/protscale/ (accessed on 28 November 2025) [35]) to generate residue-level hydrophobicity/hydrophilicity profiles (e.g., Kyte-Doolittle scale) for mapping amphipathic regions [31]. We interrogated the SMART database to identify evolutionarily conserved domains within the OsWRKY7 protein for functional annotation. We applied TMHMM 2.0 (DTU Health Tech) (available online: http://www.cbs.dtu.dk/services/TMHMM/ (accessed on 28 November 2025) [36]) to predict transmembrane helices while noting potential signal peptide overlaps in N-terminal predictions (with cross-validation considered using DeepTMHMM (available online: https://dtu.biolib.com/DeepTMHMM (accessed on 28 November 2025) [37]) for enhanced accuracy). In addition, we applied NetPhos 3.1 Server (available online: https://services.healthtech.dtu.dk/services/NetPhos-3.1/ (accessed on 28 November 2025)) to prioritize serine/threonine/tyrosine phosphorylation sites, integrating evolutionary conservation scores to contextualize functional relevance (prediction of post-translational glycosylation and phosphorylation of proteins from the amino acid sequence [38]).

### 2.3. Subcellular Localization Prediction

The subcellular localization of OsWRKY7 was predicted using the CELLO v.2.5 CELLO—SubCELlular LOcalization predictor (available online: http://cello.life.nctu.edu.tw/ (accessed on 27 November 2025) [39]). The protein sequence file of OsWRKY7 was retrieved from the NCBI database and stored for subsequent analysis. The online tool CELLO v.2.5 was accessed, and the OsWRKY7 protein sequence was submitted to this platform. Subsequent submission of the sequence enabled the prediction of the subcellular localization of the target gene.

### 2.4. Analysis of Conserved Motifs and Domains in Rice WRKY Proteins

To characterize the conserved motifs of rice WRKY proteins, we employed the MEME online tool MEME—Multiple Em for Motif Elicitation (available online: https://meme-suite.org/meme/ (accessed on 28 November 2025) [40]) with parameters configured to identify 10 motifs spanning 6–50 amino acids in length. Conserved domain annotations for these proteins were retrieved from the NCBI Conserved Domain Database (CDD) (available online: http://www.ncbi.nlm.nih.gov/Structure/bwrpsb/bwrpsb.cgi (accessed on 28 November 2025) [41]). Gene structure analysis of the rice WRKY family in chromosome 5 was conducted using the rice genome annotation file (version 7.0) from the Rice Genome Annotation Project (RGAP) Rice Genome Annotation Project (available online: http://rice.uga.edu/ (accessed on 28 November 2025) [42]). Finally, conserved motifs, domains, and gene structures were visualized using the advanced GeneStructureView tool integrated within TBtools-II [43].

### 2.5. Analysis of the Transcriptional Activation Activity of OsWRKY7

All chemical reagents, prepared media, and cloning vectors employed in this work were obtained from Coolaber Science and Technology Co., Ltd. (Beijing, China). (YM2000-1Set, YS3093, YS3091).

The transcriptional activation activity was assessed using a yeast two-hybrid (Y2H) assay, as previously described by Li et al. [44] with minor modifications. The full-length coding sequence (CDS) of *OsWRKY7*, along with its N-terminal (1–360 bp) and C-terminal (361–666 bp) CDS fragments, was used as a template for primer design. For each fragment, forward and reverse primers of 18–22 bp in length were designed, with annealing temperatures around 60 °C, and homologous arms matching the pGBKT7 vector were added to their respective ends. Using rice cDNA as the template, polymerase chain reaction (PCR) was performed to amplify the corresponding fragments, each flanked by the pGBKT7 homologous arms. These fragments were then assembled into the linearized pGBKT7 vector via homologous recombination, resulting in the construction of the recombinant vectors pGBKT7-OsWRKY7, pGBKT7-OsWRKY7-N, and pGBKT7-OsWRKY7-C. Primer sequences are listed in Table S1. These plasmids were then co-transformed with pGADT7 (carrying the transcriptional activation domain) into Y2HGold yeast competent cells using the PEG/LiAc method, in accordance with the Yeastmaker™ Yeast Transformation System 2 kit (coolaber). Transformed yeast cells were selected on SD/-Leu/-Trp (DDO) solid medium and incubated at 30 °C for 3 days. Individual colonies were subsequently inoculated into DDO liquid medium and grown at 30 °C until shake-cultured for 16–18 h until OD600 ≈ 0.8. A 10 μL aliquot of each culture was spotted onto both SD/-Leu/-Trp and SD/-Leu/-Trp/-His/-Ade (QDO) agar plates, followed by incubation at 30 °C for 3–4 days to monitor growth. Yeast cells co-transformed with empty pGBKT7 and pGADT7 vectors served as the negative control.

### 2.6. Analysis of Promoter Cis-Element and Haplotype

The 2000 bp promoter region upstream of the ATG translation initiation codon of the *OsWRKY7* gene was extracted from the Rice Genome Annotation Project (RGAP, version 7.0) database. Using the PlantCARE online platform (available online: http://bioinformatics.psb.ugent.be/webtools/plantcare/html/ (accessed on 28 November 2025) [45]), we conducted a comprehensive computational scan of this sequence to systematically identify cis-acting elements, characterizing their types, genomic distribution patterns, and putative regulatory roles. To visually resolve the spatial arrangement of these elements, TBtools-II software was employed to generate high-resolution visualizations of the predicted cis-acting elements within the promoter, enabling intuitive interpretation of their compositional diversity and positional distribution characteristics across the regulatory region.

Single-nucleotide polymorphisms (SNPs) were obtained from 10,548 rice accessions deposited in the RiceSuperPIRdb database (available online: RiceSuperPIRdb (http://www.ricesuperpir.com, accessed on 28 November 2025) [46,47]). Wild rice SNPs were sourced from our previous study [48]. Pairwise values were analyzed using Haploview 4.0 [49].

### 2.7. Analysis of OsWRKY7 Expression Characteristics

The tissue-specific expression pattern of *OsWRKY7* was predicted using the eFP website eFP Browser (available online: http://bar.utoronto.ca/efp_rice/cgi-bin/efpWeb.cgi (accessed on 27 May 2024) [50,51]).

### 2.8. RNA Extraction and Quantitative Real-Time PCR (qRT-PCR)

The rice cultivar ‘ZH11’ was selected as the experimental material for quantifying the expression of *OsWRKY7*. Two-week-old hydroponically cultivated rice variety ‘ZH11’ seedlings, grown at 28 °C, provided the experimental materials (0.5 g of samples), including leaves, stems, and roots. Total RNA was isolated using the Easy Rice RNA Rapid Extraction Kit (Non-TRIzol method, Cat# DR0408050, Hangzhou, China), and qRT-PCR was performed with the HieffqPCR SYBR Green Master Mix (Cat# 11201ES60, Shanghai, China) according to the manufacturer’s protocol, employing gene-specific primers (Table S1). Transcript levels were normalized to the *ACTIN* gene using the 2^−ΔΔCt^ method.

### 2.9. Statistical Analysis

All quantitative experiments were performed with at least three independent biological replicates. Data are presented as mean ± standard deviation (SD). Prior to parametric analysis, the normality of all datasets was verified using the Shapiro–Wilk test (*p* > 0.05), and homogeneity of variances was confirmed by Levene’s test (*p* > 0.05). One-way ANOVA with Tukey’s post hoc test was employed for multiple comparisons when more than two groups were analyzed. Statistical analyses were conducted using GraphPad Prism 9.0, with *p* < 0.05 considered statistically significant.

## 3. Results

### 3.1. Analysis of WRKY7 Structural Conservation

This study systematically identifies homologous genes of *OsWRKY7*, a key member of the WRKY transcription factor family in rice (*Oryza sativa*), and reconstructs the evolutionary history of the *WRKY7* subfamily across diverse plant lineages. We initiated our analysis by performing homology searches using the BLASTP 2.17.0 algorithm (E-value threshold = 1 × 10^−10^, maximum target sequences = 1000, coverage ≥ 30%) against the NCBI non-redundant protein database, with the OsWRKY7 protein sequence as the query. This approach successfully identified *WRKY7* homologs from over 50 representative plant species, spanning angiosperms (e.g., *Arabidopsis thaliana*, *Zea mays*, *Setaria italica*), gymnosperms (e.g., *Pinus* spp.), ferns (e.g., *Nephrolepis cordifolia*), and lycophytes (e.g., *Selaginella moellendorffii*) (Table S2).

**Figure 1:**
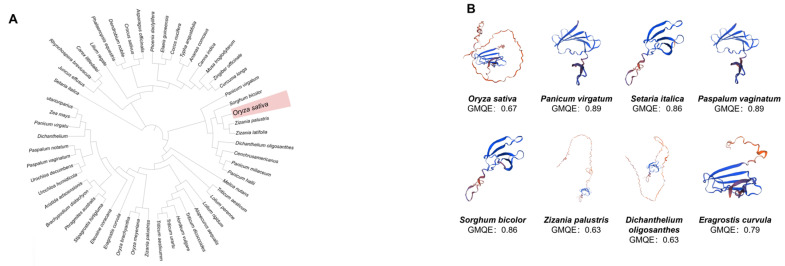
Evolutionary analysis of *WRKY7* in green plants. (**A**) Maximum likelihood-based phylogenetic analysis of WRKY7 proteins in representative species of plants. *Oryza sativa* was showen in red color. (**B**) The three-dimensional structure of the protein predicted based on the protein sequences of *Oryza sativa* and various species of WRKY7 (https://swissmodel.expasy.org/).

To elucidate evolutionary relationships, we reconstructed a neighbor-joining phylogenetic tree with 1000 bootstrap replicates using *WRKY7* protein sequences from 20 strategically selected species representing key evolutionary nodes, including basal monocots, core eudicots, and basal fern lineages. The resulting phylogeny, presented in a circular layout (Figure 1A), highlights *Oryza sativa* within a red rectangle. Our analysis reveals that the *OsWRKY7* ortholog in *Setaria italica* shares 78.6% sequence identity and clusters with rice in a basal clade. Molecular dating estimates indicate their divergence predates the monocot–eudicot split (≈160 million years ago), suggesting the *WRKY7* subfamily originated in basal angiosperm lineages, particularly within the Poales order, and has been functionally maintained across diverse plant groups throughout evolution.

To investigate structural conservation, we selected WRKY7 orthologs from a phylogenetically diverse set of species, including *Oryza sativa*, *Panicum virgatum*, *Setaria italica*, *Paspalum vaginatum*, *Sorghum bicolor*, *Zizania palustris*, *Dichanthelium oligosanthes*, and *Eragrostis curvula*. Homology models of the corresponding WRKY7 proteins were generated using the SWISS-MODEL server (https://swissmodel.expasy.org, accessed on 28 November 2025), enabling comparative analysis of conserved structural features across representative monocot and eudicot taxa (Figure 1B). This integrated (WRKYGQK sequence and zinc finger structure) approach provides valuable insights into the evolutionary preservation of critical structural determinants underlying WRKY7 function in plant biology.

**Figure 2:**
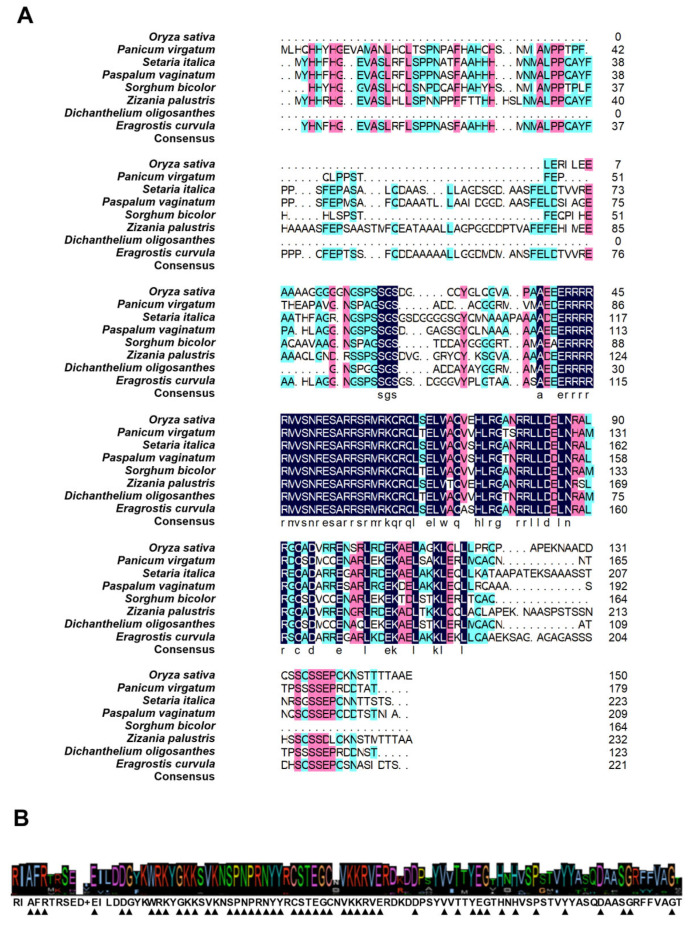
Phylogenetic and structural analysis of the WRKY7 protein across *Poaceae* species. (**A**) Multiple sequence alignment of WRKY7 amino acid sequences from eight representative *Poaceae* species: *Oryza sativa*, *Panicum virgatum*, *Setaria italica*, *Paspalum vaginatum*, *Sorghum bicolor*, *Zizania palustris*, *Dichanthelium oligosanthes*, and *Eragrostis curvula*. The alignment highlights the invariant WRKYGQK motif (indicated by the navy blue bar) and the zinc-finger-like structures, demonstrating high conservation of the core functional domains. (**B**) Schematic representation of the conserved protein motifs identified in WRKY7 proteins. Motifs were predicted using the MEME suite.

To complement our phylogenetic findings, we performed multiple sequence alignment (MSA) using ClustalW and predicted secondary structures with MEMSAT-SVM. These analyses revealed remarkable conservation in both sequence and structural features among WRKY7 proteins across all examined species. The MSA identified several highly conserved regions (gray-shaded) and core functional motifs (red-highlighted), including EILDGKYW and RNYVRCST, which showed minimal sequence variation despite moderate length differences among orthologs (ranging from 64 residues in *Dichanthelium oligosanthes* to 132 residues in *Eragrostis curvula*) (Figure 2).

Collectively, these findings establish the evolutionary origins of the *WRKY7* subfamily (basal angiosperms) and its functional conservation across the plant kingdom, providing critical evidence for further investigations into its biological roles and adaptive evolution (Figure 1 and Figure 2).

### 3.2. Physicochemical Properties and Structural Analysis of the OsWRKY7 Encoding Protein

**Figure 3:**
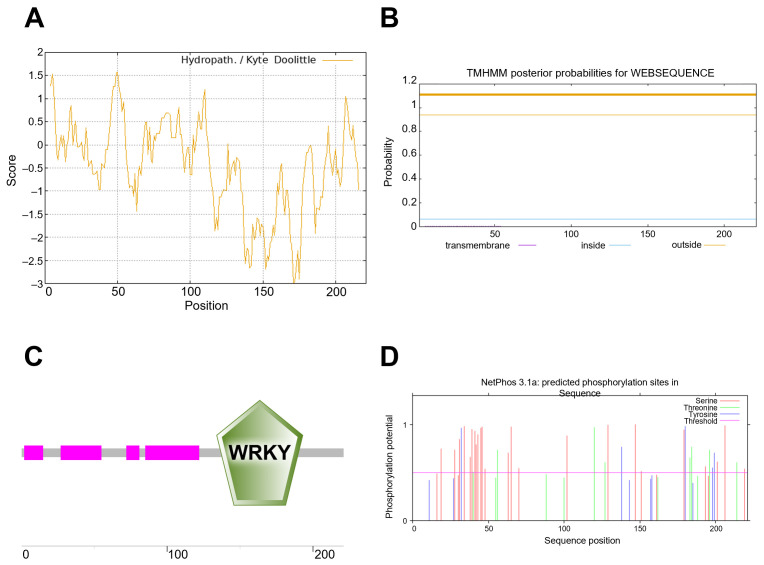
In-depth bioinformatic characterization of *OsWRKY7*. (**A**) Hydropathy profile of OsWRKY7 predicted by the Kyte-Doolittle algorithm. The plot reveals the distribution of hydrophilic and hydrophobic regions along the protein sequence, with positive values indicating hydrophobic areas (https://web.expasy.org/protscale/, accessed on 28 November 2025). (**B**) Prediction of transmembrane helices. Analysis using TMHMM or a similar tool indicates the absence of substantial transmembrane domains, suggesting OsWRKY7 is not an integral membrane protein (https://services.healthtech.dtu.dk/services/TMHMM-2.0/, accessed on 28 November 2025). (**C**) Schematic architecture of OsWRKY7 domain organization. The diagram depicts the relative positions of key domains, including the N-terminal region, the central WRKY domain, and the potential zinc-finger motif (https://dtu.biolib.com/DeepTMHMM/, accessed on 28 November 2025). (**D**) Forecast of potential phosphorylation sites. NetPhos or a similar predictor was employed to identify serine (S), threonine (T), and tyrosine (Y) residues that are likely targets for post-translational modification by kinases (https://services.healthtech.dtu.dk/services/NetPhos-3.1/, accessed on 28 November 2025).

The OsWRKY7 protein is encoded by a 711 bp open reading frame and comprises 221 amino acids, with a predicted molecular weight of 23.17 kDa and a theoretical isoelectric point (pI) of 6.59. Biochemical characterization classified OsWRKY7 as an unstable hydrophilic protein, supported by an instability index of 52.76 and a grand average of hydropathicity (GRAVY) score of −0.441 (Figure 3A). Structural analysis using TMHMM confirmed the absence of transmembrane domains, indicating OsWRKY7 is not membrane-associated (Figure 3B). Domain architecture analysis revealed a canonical WRKY DNA-binding domain spanning residues 134–193, consistent with the conserved structure of plant WRKY transcription factors (Figure 3C). Post-translational modification prediction via NetPhos 3.1 identified 42 potential phosphorylation sites, including 28 serine, 13 threonine, and 11 tyrosine residues (Figure 3D), suggesting OsWRKY7 may undergo extensive regulatory phosphorylation by Ser/Thr/Tyr kinases. Subcellular localization prediction using CELLO v.2.5 indicated nuclear enrichment of OsWRKY7, supporting its predicted function as a transcription factor (Figure S1).

### 3.3. Structural Characteristics of the WRKY Protein Family Domains and Genes in Rice

**Figure 4:**
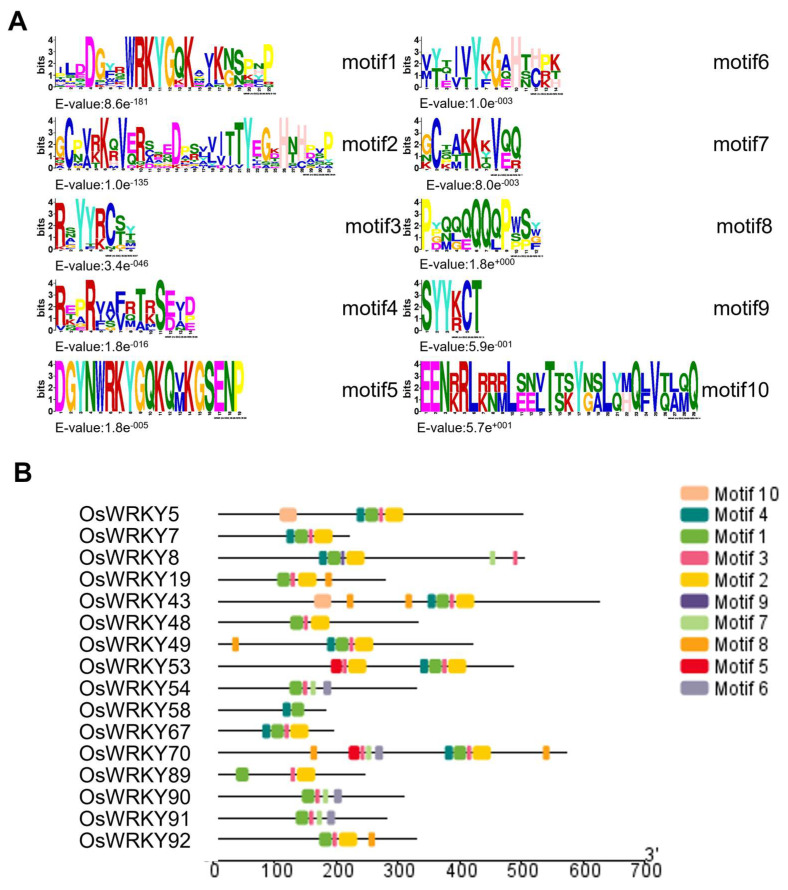
Analysis of conserved motifs in the rice WRKY gene family. (**A**) Sequence logos of individual conserved motifs. The logos depict the amino acid sequence and conservation level for each motif, with the height of letters representing the information content at each position. The highly conserved ‘WRKYGQK’ signature within the core WRKY domain is explicitly visible. (**B**) Schematic distribution of conserved motifs across OsWRKY proteins. Each colored box represents a distinct motif (e.g., Motifs 1–10) identified by MEME analysis. The connecting black lines, scaled to protein length, illustrate the order and arrangement of these motifs within each protein.

Our analysis identified ten conserved motifs, designated motif 1 to motif 10 in order of increasing E-values (Figure 4A). Notably, motif1 was present in all WRKY proteins encoded by chromosome 5-localized genes (Figure 4B), indicating that it represents a core, evolutionarily conserved signature of OsWRKY proteins. Furthermore, most WRKY family members contained motifs 1 through 7, reflecting substantial sequence conservation and suggesting potential functional redundancy among rice WRKY factors. Together, these findings support the hypothesis that *OsWRKY7* may perform biological roles analogous to those of other WRKY family members in rice.

### 3.4. Transcriptional Activation Analyses of OsWRKY7

**Figure 5:**
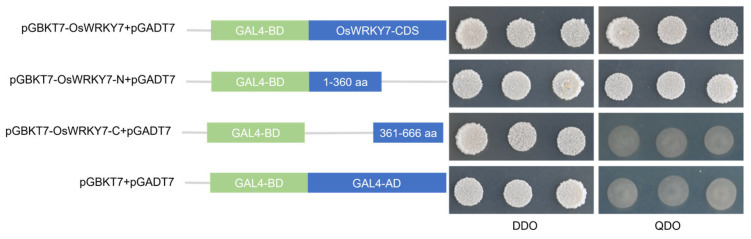
Transcriptional activation assay of full-length and truncated OsWRKY7 proteins in yeast. The full-length (OsWRKY7, 1–666 aa) and truncated segments, comprising the N-terminal domain (OsWRKY7-N, 1–360 aa) and the C-terminal domain (OsWRKY7-C, 361–666 aa), were fused to the GAL4 DNA-Binding Domain (BD) in the pGBKT7 vector. Yeast transformants were spotted on synthetic dropout (SD) media lacking Leu and Trp (DDO) to confirm transformation efficiency and on selective media lacking Leu, Trp, His, and Ade (QDO) supplemented to assess transcriptional activation.

To further investigate the transcriptional activation potential of OsWRKY7, we conducted a functional assay in yeast cells. The results demonstrated that OsWRKY7 possesses strong transcriptional activation activity in yeast (Figure 5). Domain-deletion analysis further revealed that this transactivation capability is primarily mediated by its N-terminal domain (Figure 5).

### 3.5. Analysis of OsWRKY7’s Promoter Elements and Haplotype

**Figure 6:**
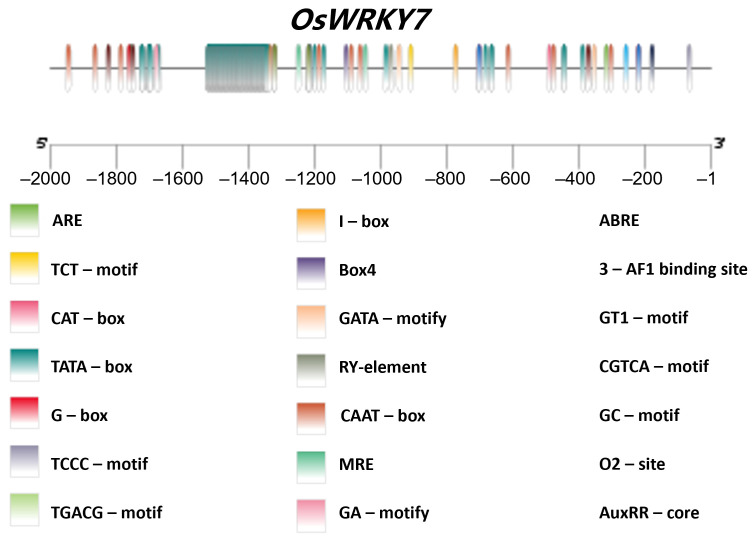
Visualization of the promoter element analysis of *OsWRKY7*. Schematic representation of the distribution of predicted cis-regulatory elements within the 2.0 kb promoter region upstream of the *OsWRKY7* translational start site. The analysis was performed using PlantCARE (PlantCARE, a database of plant promoters and their cis-acting regulatory elements). Key identified elements are color-coded and include core promoter elements (TATA-box, CAAT-box), hormone-responsive elements (e.g., for abscisic acid, methyl jasmonate, and auxin), stress-responsive elements (e.g., for drought), and light-responsive elements.

To characterize the cis-acting elements within the promoter region of *OsWRKY7*, we extracted the 2.0 kb genomic sequence upstream of its translational start codon from the Rice Annotation Project Database (RAP-DB) (Figure S2). Subsequent analysis of these promoter sequences was performed using the online tool PlantCARE (Table S3). Our results revealed that, in addition to the canonical TATA-box and CAAT-box core promoter elements, the *OsWRKY7* promoter contains multiple cis-acting regulatory elements associated with hormone signaling and abiotic stress responses. Specifically, these include elements responsive to abscisic acid (ABA), methyl jasmonate (MeJA), auxin, and drought stress. Notably, we also identified light-responsive cis-acting elements within the promoter, suggesting that *OsWRKY7* may function in integrating hormone-mediated, abiotic stress, and light perception signaling pathways during plant growth and development (Figure 6).

To investigate the genetic diversity and evolutionary dynamics of the rice *OsWRKY7* gene, we conducted a haplotype analysis using Haploview software 4.0. The following parameters were evaluated for each polymorphic site: Position (physical location on the chromosome), ObsHET (observed heterozygosity), PredHET (predicted heterozygosity under Hardy–Weinberg equilibrium), HWval (*p*-value from Hardy–Weinberg equilibrium test), %Geno (genotyping success rate), FamTrio (number of family trios analyzed), MendErr (number of Mendelian errors detected), MAF (minor allele frequency), and Alleles (nucleotide variants observed). Values preceded by ‘<’ indicate measurements below the detection threshold. We focused on single-nucleotide polymorphisms (SNPs) within the *OsWRKY7* locus and filtered SNP data from a natural rice population with a minor allele frequency (MAF) threshold of ≥0.05 (Table S4). This analysis identified only two well-differentiated haplotypes, designated Hap1 and Hap2, which were stably present across accessions. However, the limited number of haplotypes (n = 2) substantially restricts the ability to perform subsequent evolutionary and functional analyses, as the low genetic diversity provides insufficient resolution for robust statistical inference (Figure S3).

### 3.6. The Expression Patterns of OsWRKY7 in Different Tissues

**Figure 7:**
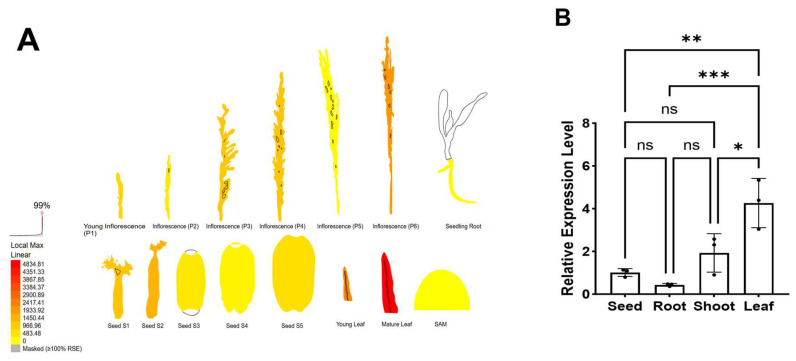
The expression patterns of *OsWRKY7* in different tissues. (**A**) In silico analysis of *OsWRKY7* expression across different rice tissues and developmental stages, obtained from the Rice eFP Browser (https://bar.utoronto.ca/efprice/cgi-bin/efpWeb.cgi). Data indicate that *OsWRKY7* is ubiquitously expressed in seeds, roots, leaves, inflorescences, and shoot apical meristems (SAM), with the highest transcript abundance detected in mature leaves. (**B**) Experimental validation of *OsWRKY7* expression by qRT-PCR in the cultivar ZH11. Relative expression levels were measured in seed, root, shoot, and leaf. Data were reported relative to the seeds, which were assigned as 1.0. Values were shown as means ± standard deviation (n = 3) and analyzed by one-way ANOVA followed by Tukey’s multiple comparisons test (*p* < 0.05). *, **, ***, and ns indicate statistical significance levels: * *p* < 0.05, ** *p* < 0.01, *** *p* < 0.001, and ns (not significant) for *p* ≥ 0.05, respectively.

By analyzing the expression of *OsWRKY7* in different rice tissues through the rice eFP online website (https://bar.utoronto.ca/efprice/cgi-bin/efpWeb.cgi, accessed on 28 November 2025) (Figure 7A), it was found that *OsWRKY7* is expressed in rice seeds, roots, leaves, inflorescences, and shoot apical meristems, with the highest expression in mature leaves. This indicates that the expression of *OsWRKY7* has significant spatiotemporal specificity and may play its biological function in different parts of rice. Furthermore, we performed quantitative real-time PCR (qRT-PCR) to monitor *OsWRKY7* expression across various rice tissues (Figure 7B). The results aligned with bioinformatic predictions: *OsWRKY7* expression was highest in leaves, while no significant differences were observed among seeds, roots, and stems. This tissue-specific expression pattern corroborates the computational predictions and substantiates the proposed functional diversification of *OsWRKY7* in different plant tissues.

## 4. Discussion

This study provides a systematic characterization of the transcription factor *OsWRKY7* in rice, elucidating its evolutionary origin and functional properties. Our findings demonstrate that the WRKY7 subfamily originated in basal angiosperms and exhibits remarkable conservation in both protein sequence and three-dimensional structure across monocot and eudicot lineages, suggesting its fundamental and non-redundant biological role. *OsWRKY7* encodes a nuclear-localized, unstable hydrophilic protein with strong transcriptional activation capacity mediated by its N-terminal domain. Promoter analysis revealed the presence of multiple cis-acting elements responsive to abscisic acid, methyl jasmonate, and drought stress. Furthermore, expression profiling showed predominant accumulation in mature leaves with distinct spatiotemporal specificity. Collectively, these results indicate that *OsWRKY7* appears to integrate hormonal and environmental signals to modulate stress adaptation and developmental processes in rice. This functional role supports its potential as a candidate gene for molecular breeding of stress-resilient varieties.

*OsWRKY7* exhibits a dual pattern of “highly conserved commonality” and “subfamily-specificity” in its evolution and homology compared to other rice WRKY family genes. Similar to defense-related members such as *OsWRKY53* [52], *OsWRKY7* possesses the complete WRKYGQK sequence and zinc finger structure, which are essential for binding to the W-box cis-element in target genes, reflecting a core functional commonality within the family. However, in contrast to certain WRKY genes (e.g., OsWRKY45 [53]) that have undergone artificial selection during domestication, resulting in novel alleles, *OsWRKY7* displays remarkable evolutionary stability. This conservation strongly suggests that *OsWRKY7* may fulfill a more fundamental and core physiological role, the integrity of which is critical enough to be maintained under natural selection, thereby rendering its function non-redundant and irreplaceable by other WRKY members.

Furthermore, the full-length OsWRKY7 protein exhibits typical transactivation activity in a yeast system, a characteristic shared with several documented rice WRKY members such as *OsWRKY95* [54] and *OsWRKY53* [55]. This functional similarity suggests evolutionary conservation of regulatory mechanisms among these transcription factors, likely linked to their central roles in disease resistance and abiotic stress responses. Notably, analogous to *OsWRKY26* [56], the transactivation domain of *OsWRKY7* was mapped to its N-terminal region. Based on this finding, we generated a truncated version of *OsWRKY7* lacking the N-terminal activation domain. This construct can serve as bait in subsequent yeast one-hybrid or two-hybrid screens to systematically identify proteins that interact with *OsWRKY7*. This approach will provide critical experimental groundwork for elucidating the regulatory network and downstream signaling pathways governed by *OsWRKY7*.

The regulatory potential of *OsWRKY7* is further emphasized by its promoter architecture, which is enriched with cis-acting elements responsive to abscisic acid, jasmonic acid, auxin, and drought stress. This structural feature parallels the regulatory patterns observed in other rice WRKY members: for instance, *OsWRKY45* expression is strongly induced by the SA analog BTH [57], while *OsWRKY72* is upregulated by ABA and PEG-mediated drought simulation [58]. At the post-translational level, *OsWRKY53* serves as a substrate for *OsMPK3*/*OsMPK6*, and phosphorylation of its N-terminal region significantly enhances its transactivation activity [59,60]. In light of these conserved regulatory mechanisms, we propose that *OsWRKY7* likely functions as a key transcriptional decoder of developmental and environmental signals, potentially orchestrating resource allocation between stress resilience and reproductive development—a fundamental trade-off in rice.

By elucidating the strong purifying selection and pronounced haplotype bottleneck that define *OsWRKY7*’s evolutionary distinctiveness, this study reveals its non-redundant biological functions [61,62,63,64]; furthermore, the discovery of its N-terminal transcriptional activation mechanism—distinct from conventional WRKY domain-centric models—establishes a unique molecular regulatory paradigm for this gene. Most significantly, its seed-specific expression pattern combined with cis-regulatory complexity underscores specialized roles in developmental regulation [65,66,67,68]. These findings systematically elevate *OsWRKY7* from an uncharacterized WRKY member to an evolutionarily constrained regulator, not only opening new avenues for deciphering rice transcriptional networks but also enabling the utilization of its genetic stability for trait consolidation in breeding programs.

## Data Availability

The original contributions presented in this study are included in the article/Supplementary Material. Further inquiries can be directed to the corresponding author.

## Supplementary Materials

The following supporting information can be downloaded at https://www.mdpi.com/article/10.3390/life15121852/s1, Figure S1. Prediction of OsWRKY7 subcellular localization. Figure S2. Analysis of the *OsWRKY7* promoter. Figure S3. Haplotype analysis of *OsWRKY7*. Table S1. Primers and sequences in this study. Table S2. Protein sequences of OsWRKY7 homologs from diverse plant species. Table S3. Cis-acting elements in the *OsWRKY7* promoter region. Table S4. Haplotype analysis of *OsWRKY7*.
