# Supplementary material for: Functional Characterization of OsWRKY7, a Novel WRKY Transcription Factor in Rice
Source: Life (Basel). 2025 Dec 2;15(12):1852. doi: 10.3390/life15121852 (PMC12734347; doi:10.3390/life15121852)
Supplement: Supplementary file 1 [file life-15-01852-s001.zip › life-3994022-supplementary.pdf]

CELLO RESULTS

SeqID: LOC_Os05g46020.1

Analysis Report:

SVM	LOCALIZATION	RELIABILITY
Amino Acid Comp.	Nuclear	0.511
N-peptide Comp.	Nuclear	0.822
Partitioned seq. Comp.	Chloroplast	0.506
Physico-chemical Comp.	PlasmaMembrane	0.457
Neighboring seq. Comp.	Nuclear	0.511

CELLO Prediction:

Nuclear	2.144	*
Chloroplast	1.229	
PlasmaMembrane	0.556	
Extracellular	0.342	
Mitochondrial	0.296	
Cytoplasmic	0.204	
Vacuole	0.096	
Peroxisomal	0.049	
ER	0.031	
Cytoskeletal	0.019	
Golgi	0.019	
Lysosomal	0.014	

Figure S1 Subcellular Localization Prediction of the OsWRKY7 Protein by CELLO 2.5. The figure presents the prediction scores for various subcellular localizations. A higher score indicates a greater probability of localization. The nucleus received the highest score (2.144), marked with an asterisk (*) as the primary predicted location, suggesting that OsWRKY7 is most likely a nuclear protein.. (https://cello.life.nctu.edu.tw/).

OsWRKY7 Promotor Sequence

GCTGGCCGAACATGGGCGAATAGAAGTAAATATTGATTATTGACAGTGATTGTT
AGTATCAAATGAAACAGTTTTGGTAGGCATAGCCGCATAGGCATGAAATTTTCG
TTATCATATTTTTTAAATTGTTGAATGGTGCATTTGCACTAAAACTTTCTACTTCC
TCCGTCTAAAATAAACTTAACCTTGAAGAGGATGGGACCATCCTGGATAAAGAA
TTTGGACAACCTCCTTTGTCGAGATTCCCCTCTTAGGTT/GGTTAAGTTTTTTTT
CGACGGAGTGAGTATATAATAGCTGCTTTAATGTATTATATAAAATATTTTTAAAG
TTTATCTATAGTAGTAATAATTAAAATGTAATTAACTATGAGCTAATGACATTGTTG
TTTTTTTCCATAACTTCATCTCCATCTTAAGTAGAAACGAACACTACCTAAGTGG
GTAGTGTCTCATGAGAAAAATTCTTTAGATGAAATTCTAGCGAAGTTATATATATA
TATATATATATATATATATATATATATATATATATATATATATATATATATATATATA
TATATATATATATATATATATATATATATATATATATATATATATATATATATATATA
TATATATATATATATATATATATATATATATATATATATATGTGAGATTATGTGGAA
TTTG/TGACGAAATTCATGAGTTTTGAGCTAATTATTCAATGAGCCATGTTCCGA
CCGGTGAACTTTCATAAACAACTTAGGTTGTGTTAAATCCAGATGTAAAGTTTT(
GACG/G(ACG)TG/ACGTG)TCACATCGGATATACGAATACATATTTGAAGTATTAA
ATATAGTCTAATAACAAAATAAATTACAGGATCCACCTGTAAACCGCGAGATGAA
TTTATTAAGCCTAATTAATCCATCATTAGCAAATGTTTACTGTAGCACCACATTGT
CAAATCATAGCGCAATTAGGTTTAAAAGATTCGTCTCGTAATTTACATGCAATCT
ATGTAATTAGTTATTTTTTCGTCTATATTTAATACTCCATGCATGTGTTAAACATTT
AATGTGATAGGGTGTGAAATTTTGCTAAGGGATAAAAAGGATCTTACAGGCATA
AAATACATGACTAATAATGGCTGAAGCGAAAAAGGATAAATACACCAGATTTAA
CTGTGGTAGCTACAATATCTCTAACTTTCCTGTCAATAGGTTTGATTAGACTCG
GGCAAAGCAGGAACACACCTGATCCAAGACCCGATACCTCTTGGACAATCGG
AGCACGATAGTATTAGCAGGCAAAAATCAACGCC(CACGT/CACGT)CGGTGTC
GGGTGCGTATTTAAAAACATCGATAATATATGCATGGCTAGATAACTAAACAGAG
ATTATGAATCAGCACATATGATTTGGGAGCATCATGTCAGACATGTCGAACACA
TGCCCGTGCCTATCGAACACAGTATCCGTGGGTAAAATCGCTATCGCTGCTCA
GTCCAAAGTAGACGGAGCTAAAATAAAATAAAGAGAGATGCCACTCATTTAATT
TGTTTTATTTTAGATCGAATTTAAACTGATATAAGCGTCTGAGTTTAAATTAAGTG
AGTGCATATTTAATGAAAATAAATAAACTATATCTAAAAAATTTTCTTAACCGGTT
CTTGACTATCCTATCTCCTAGCTTCACCCCCTCCCAAAATTTCTTGGTTTAGCC
ACACCAACCAACTCGCAGGGAACCCAAACAAAGAAAGCGGCTTGAACCGGAT
GGACCGTGGGTGTGTGAGAAGGCCCCCACGGCAACTACGTGGCGAAGAGG
CGAAGACCGCACCCCGTCGAAGTCTTTCCCCCGGAGACATTTCCGCCATGCT
GCTAAAATAAGTGGGCACGCCGTGCGCGCGTGCGTGCGTTCGAGCGGCGGC
GCGCGCGCGGCAACTCCCGCACCCGCTCCGAACCTTCTCCCTCCTCACGAA
GCTCACCGCGAAGCCGGCAAAATCCTCCCGGAGAGAGAGACACAAACACA

Motifs

ARE MRE ABRE GA-motif Box 4 CAAT-box G-box RY-element GC-motif
CGTCA-motif/TGACG-motif GATA-motif O2-site CAT-box I-box AuxRR-core
GT1-motif 3-AF1 binding site G-box TCCC-motif TCT-motif

Figure S2 Analysis of the *OsWRKY7* promoter. Different colored letters represent distinct motif types.

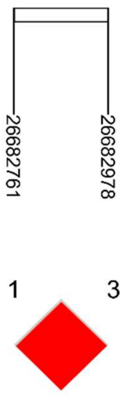

Figure S3 Haplotype analysis of OsWRKY7.

Table S1 Primers and sequences in this study

Primer name	Forward primer(5'-3')	Reverse primer(5'-3')
BD-OsWRKY7-N	GGCCATGGAGGCCGAATTCATGGCGGCAGTCGGCGCG	GCTGCAGGTCGACGGATCCCGTCCGCGGCCTCTCCAC
BD-OsWRKY7-C	GGCCATGGAGGCCGAATTCGAGCGGATCGCGTTCCGG	GCTGCAGGTCGACGGATCCTTAATTGAGAGAACCTGGCGG
BD-OsWRKY7	GGCCATGGAGGCCGAATTCATGGCGGCAGTCGGCGCG	GCTGCAGGTCGACGGATCCTTAATTGAGAGAACCTGGCGG
qRT-*OsWRKY7*	ACCCAAGGAACTACTACCGGT	TCACGTGGTTGTGCGTCC
qRT-*actin*	ATCCTTGTATGCTAGCGGTCGA	ATCCAACCGGAGGATAGCATG

Table S2 Protein sequences of OsWRKY7 homologs from diverse plant species

name	sequence
>*Alopecurus aequalis*	MAAVGAAPVLYQQSALDATCFFSSMSSHFSNGHGGVGSTTSSSPSPASSFSAALGAVPVQPAVADPAGQFDISEYLFDDSVFAAPPPSGTLVPGANATTAPHARSAAEATAVAERPRTERIAFRTRSVIEILDDGYKWRKYGKKSVKNSPNPRNYYRCSTEGCDVKKRVERDRDDPAYVVTMYEGTHSHVSPSTVYYASQDAASGRFFVAGTHPPPGSLD
>*Ananas comosus*	PPAANPTSGFDLSDFIFFDEGSTPPTSFAQLGTGAITPQPATESAYAGQSSGRSVPASGSSTTSGIRAEVERPRTERIAFRMKSEVEIVDDGFRWRKYGKKSVKNSPNPRNYYRCSTEGCSVKKRVERDKDDPSYVITTYEGVHNHMSPGVVYYTTQDAASGRYFVAGCQIPPGS
>*utariori parius*	MAAVGALPVLYHHPAPAGDAASMSSYFSHGGSSTTSSSASSFSAALAPTTTALAEQFDISEFLFDDARAAGAPGVFADGAAPVVVSDAAAAGGGAISAAAGSAAAAAEAVPERPRTERIAFRTRSEIEILDDGYKWRKYGKKSVKNSPNPRNYYRCSTEGCNVKKRVERDKDDPSYVVTTYEGTHNHVSPSTVYYASQDAASGRFFVAGTQPPGSLN
>*Aristida adscensionis*	MAAVGARPVLYHHPAPAGDAAFSAMSSFFSHGGSSTSSSPASSFSAAAAAAALGGPASATAAPLPPPLAADPAAQFDISEYFLDDGVFAAPPDDVSPPLAPADGAAGAGAIAHSARSTAEAPPERPRTERIAFRTRSEIEILDDGYKWRKYGKKSVKNSPNPRNYYRCSTEGCSVKKRVERDKDDPSYVVTTYEGRHNHVSPSTVYYASQDAASGRFFVAGTQPP
>*Asparagus officinalis*	LDLSDYILTDEVPTECSGPASTAQLPIQGRLASSIAEASNAASSHRANNYVDSSSGGGGGGDNHNTRSSREGHRVAFRTKSEVEIMDDGFKWRKYGKKSVKNSPNPRNYYRCSTEGCSVKKRVERDRNEPSYVITTYEGMHNHTSPDVVYYATQDLESGRFVVSG
>*Brachypodium distachyon*	FSPALPTQPPPVTDPAAQFDISEYLFDDGIFAAATDAAAPPSGAAVAAAMDGVGASAVAALGRSPADQQQQQAAVERPRTERIAFRTRSEIEILDDGYKWRKYGKKSVKNSPNPRNYYRCSTEGCSVKKRVERDRDDPSYVVTTYEGTHSHVSPSTVYYASQDAASGRFFVAGTQPPGSLH
>*Canna indica*	FDISDFITFDQPDQFAPAAAHQSPPRAMDAGGYFGNFSNDNTHTLRNITGVESGGGRRDETSRIAFRMKSEVETLDDGYKWRKYGKKSVKNSPNPRNYYRCSTEGCSVKKRVERDREDPSYVITTYEGTHNHLSPGVVYYASQDTVSGRYYVAGCQMP
>*Carex littledalei*	FGTGLTGDVNSDQPLPFDPSEYLRDDLSTHSAFSQFMDNSLFQAVQSNGVNLPVDGGNSCVSKITGMNKPRTERIAFRMKSEVEIIDDGFKWRKYGKKSVKNSPNPRNYYKCSTEGCSVKKRVEKEKDDPSYVITTYEGIHNHVSPSMVYYTSQDAGSGQYYVSGYQISPGS
>*Cenchrus americanus*	WTTAEQVVVPDASGYAHARGVAAVVGEGTTARTTDSRIAFRVRSEEEVLDDGYKWRKYGKKSVKNSPNPRNYYRCSTEGCSVKKRVERDKDDPSYVVTMYEGVHNHVSPGTVYYATQDAASGRFFVAGMHQFG
>*Cocos nucifera*	MAAVGAPLHGPANMASYPSHAGQGGGSCDDFESDQSAAFDLSDYILLDEGVAPASFGQPETAVPPTVDVGQTSETNLAASGSGGGGTRRGGVERPRTAEGSRIAFRTRSEVEILDDGFKWRKYGKKSVKNSPNPRNYYRCSTEGCSVKKRVERDKEDPSYVITTYEGTHNHMSPSLVYYTTQDSASGRFYVAGCELPPGS

>*Crocus sativus*	FDLSDYILLDDDDECILLPTSMEQPQEQVSPVAVVADNIGISNNISNSVVEAGGGGRGFRVAFRTKSEVEVLDDGFKWRKYGKKSVKNSPNPRNYYRCSTEGCSVKKRVERDRDDPSYVITTYDGIHNHTSSDVVYYATQDDISGQFVVSG
>*Curcuma longa*	SSTTTASAAFGGLEAVAMATPAAHGPVQDQFQFGSLNQPPPANLTINTEIVAGNSSNQMGGVARRGETSRIAFRMKSEVEVPDDGFKWRKYGKKSVKNSPNPRNYYRCSTEGCCVKKRVERDRDDPSYVITTYDGTHNHMSPGVVYYTTQDSVSGRFYVAGVQ
>*Dendrobium nobile*	DYEDSSFDLSDYFLADVDPAATPAQPELAAAVPMVPSSEQNLVPPAVTGNNIRDALPPLPRMEEGSRIAFRTQTDEENLDDGFKWRKYGKKSVKNSPNPRNYYRCSTEGCLVKKRVERDNEDPSYVITTYEGIHNHSSPGVVYYATQDSVSGRFHLSGSQVQG
**>*Dichanthelium oligosanthes***	**TDRIAFRVRSEEEVLDDGYKWRKYGKKSVKNSPNPRYNFTFGINAFKLIDYYRNYYRCSTEGCNVKKRVERDKDDPSYVVTMYEGVHNHVSPGTIYYATQDAASGRFFVAGMHQLG**
>*Elaeis guineensis*	FDLSDYILLDEASFGQPAESGAPPMVDVGQTSESNLAASGSSRTSRGGVERPRTAEGSRIAFRTKSEVEILDDGFKWRKYGKKSVKNSPNPRNYYRCSTEGCSVKKRVERDKEDPSYVITTYEGXHNHMSPGVVYYTTQDSTSGRFYVAGCELPPGS
>*Eleusine coracana*	MAAVGARPVLYQYHHAGDDALSSMSSYFSQGGSSTSSSSASSFSAALAPAPIPPPIGAAADPGAAQFDISEYLFDDVAQGVFGAAPDAPPPAQHVVQAPDSGAAAAGAGAAAAQNARSAAEAMPERPRTERIAFRTKSEIEILDDGYKWRKYGKKSVKNSPNPRNYYRCSTEGCNVKKRVERDKDDPAYVVTTYEGTHNHVSPSTVYYASQDAASGRFFVAGTQPPGSLN
**>*Eragrostis curvula***	**MAAIGARPVLYHHHPAPVAGDVNASFSSMSSYFSHGGSSTSTSSAASSFSAALGAPAPPLVADPSAAQFDISEYLFDDAAQQGVFAAPHAAADAPPPPPPADSSSHGGAGASAAARSAAEALPERPRTERIAFRTRSEIEILDDGYKWRKYGKKSVKNSPNPRNYYRCSTEGCNVKKRVERDKDDPSYVVTTYEGTHNHVSPSTVYYASQDAASGRFFVAGTQPPGSLN**
>*Hordeum vulgare*	MAAVGAAPVLYQQQAQAVGDACFFSSMSSYFSNEAISSTSSSPASSFSAALGATPPAAPAIIPDPASQFDISEYLYGDGPLAAPLPPVGAAVASSATAVPARSAAESAAAVERPRTERIAFRTRTEIEILDDGYKWRKYGKKSVKNSPNPRNYYRCSTEGCSVKKRVERDRDDPAYVVTTYEGTHSHASPSTVYYASQDAASGRFFVAGTHPPPGSLN
>*Juncus effusus*	YMANYGSRSESGSVSFTAPTTGDLHSDQQALPFDLSEYILMDEGSAPTGTNFMHGGGTSSTVPVIQGGISAPLMETGVFQAGPTSSSGACTSVTAASGAMERPRTERIAFRMISEVEIIDDGFKWRKYGKKSVKNSPNPRNYYRCSTEGCSVKKRVERDKDDASYVITTYEGVHNHVTPGAVYYTTQDAASGRYFVAGMQIPPGS
>*Lilium regale*	FDITDCVTLDDGPLIEFPPIAQRSIPNLPCSSSSRVEVEKEKAGENHRIAFRTRSEVDIMDDGYKWRKYGKKTVKNSPNPRNYYRCSTDGCSVKKRVERARDDPSYVITTYEGTHNHTSPSVVYYTAQDDDSGRFFVSGCQ
>*Lolium rigidum*	KIAFRTRSEEEVLDDGYKWRKYGKKSVKNSPNPRNYYRCSTEGCSVKKRVERDRDDTNYVVTMYEGVHNHASPGTIYYASRDPATGRFFVAGMHSQG

>Melica nutans

MAAVGAPPVLYQQPAPGAGDACLFSMSSYFSHGGISSTATTSSSSTSGFSAAAALGAT
PPIVDPSAAQFDISELFFDDCTAGVFAPAPPDVPHVVVPDGATHGAAGATATATATT
LARSAAEAAVERPRTERIAFRTRSEVEILDDGYKWRKYGKKSVKNSPNPRNYYRCST
EGCSVKKRVEREKDDPSYVVTTYEGTHNHVSPSTVYY

>Musa troglodytarum

TPLHADAAAPAFDLSDFIMFDQAEFAPPSFGPEPPVVPPMVDGGRSSDQNSNLSNITI
NSGDMTVNARSTGVEGERREETSRIAFRMKSEVEVVNDGFKWRKYGKKSVKNSPN
PRNYYRCSTEGCSVKKRVERDREDPSYVITTYEGIHNHMSPGVVYYTTQDSVSGRYY
VAGCQVP

>*Oryza sativa*

**MAAVGAHAAVYHHPVSGLSAPAGDAAYSMSSYFSHGGSSTSSSASSFSAALAA
ATTPPLPDPSGSQFDISEFFFDDAPPTAVFNGAPTAALPDGAAANATRSAAEAVP
APAPAAVERPRTERIAFRTKSEIEILDDGYKWRKYGKKSVKNSPNPRNYYRCSTE
GCNVKKRVERDKDDPSYVVTTYEGTHNHVSPSTVYYASQDAASGRFFVAGTQP
PGSLN**

>Oryza brachyantha

MAAVGAHALLYHHPVVSGDGHSMSSYFSHGGSSTSSPASSFSAALGAATTPPLADPS
GAAQFDISEFFFDDAPPAPAAVFNGPPAAVLPDGAAASAAAAHATRSSSEVVPAPAV
ERPRTERIAFRTKSEIEILDDGYKWRKYGKKSVKNSPNPRNYYRCSTEGCSVKKRVER
DKDDQSYVVTTYEGTHNHVSPSTVYYASQDAASGRFFVAGTQPPAGSLN

>Oryza meyeriana

VPDAAAAGYPRSVAAAVVAGEGSARTTDRIAFRTRSDDEILDDGYKWRKYGKKSVK
NSPNPRNYYRCSTEGCNVKKRVERDKNDSRYVVTMYEGIHNHVCPGTVYFAAQDA
ASGRFFVAGMHHP

>Dichanthelium oligosanthes

MAAVGARPVLYHHPAPAGDAASMSSYFSHGGSSTSSSASSFSAALGAAPAPPLADQF
DISEFLFDDGAGGGAGVPAPGVFAAAPDGALPPAAGSAISAAAHTARSAAEATPER
PRTERIAFRTRSEIEILDDGYKWRKYGKKSVKNSPNPRNYYRCSTEGCNVKKRVERD
KDDPSYVVTTYEGTHNHVSPSTVYYASQDAASGRFFVAGTQPPGSLN

>*Panicum virgatum*

**MAAVGARPVLYHHPAPAGDAASMSSYFSHGGSSTSSSASSFSAALAAAAAPPPP
PLADQFDISEFLFDDGAGAAAAPGLFAAAADRGAPPPVAPAAGSAISAPAHAAR
SAAEAVPERPRTERIAFRTRSEVEILDDGYKWRKYGKKSVKNSPNPRNYYRCSTE
GCNVKKRVERDKDDPSYVVTTYEGTHNHVSPSTVYYASQDAASGRFFVAGTQP
PPGSLN**

>Panicum hallii

AMAYHHPCDGGGLAASAFYGGASGPAAALFTGLAPPRRQPDAAFECLSKEDVSSV
VPGTFGTPPPRMPVEQAVPDASGYAHLARGTAAVAGAEGSSTRTTDRIAFRVRSEEE
VIGDGYKWRKYGKKSVKNSPNPRNYYRCSTEGCNVKKRVERDRDDPSYVVTMYEG
VHNHVSPGTIYYATQDAASGRFFVAGMH

>Panicum miliaceum

TDRIAFRVRSEEEVLGDGYKWRKYGKKFVKNSPNPRNYYRCSTEGCNVKKRVQRDR
DDPSYVVTMYEGVHNHVKPGTIYYATQDAASGRFFVAGMH

>Panicum virgatum

TDRIAFRVRSQEEVLDDGYKWRKYGKKSVKNSPNPRNYYRCSTEGCNVKKRVERDK
DDPSYVVTMYEGVHNHVSPGTIYYATQDAASGRFFVAGMH

>*Paspalum notatum*	MAAVGARPVLYHHPAPAGDAAASMSSYFSHGGSSTSSSSASSFSAALAAAAVPAPPTLAEHFDISEFLFDDGAGAGVVAAPPGVFPAAPDGAPPPAVPDGAAVAAAGALSAAAAHHPARSGAAAEAVPERPRTGRIAFRTRSEIEILDDGYKWRKYGKKSVKNSPNPRNYYRCSTEGCNVKKRVERDKDDPSYVVTTYEGTHNHVSPSTVYYASQDAASGRFFVAGTQPPASFN
**>*Paspalum vaginatum***	**MAAVGARPVLYHHPAPAGDAASMSSYFSHGGSSTSSSASASSFSAALAAAPAPTLAEHFDISEFLFDDGAGAVAAPGVFAAAPDGAPVVPDGAAVGGGAVSAAAAHPARSAAAADAVPVPERPRTERIAFRTRSEIEILDDGYKWRKYGKKSVKNSPNPRNYYRCSTEGCNVKKRVERDKDDPSYVVTTYEGTHNHVSPSTVYYASQDAASGRFFVAGTHPPASFN**
>*Phalaenopsis equestris*	SSFDLSEYFLADEDAPPPHALPDLSAAAPTVHSSEQSLFPPAVTKDNNRDTLPILPRMEEGSRIAFRIQTDVENLDDGYKWRKYGKKSVKNSRNLRNYYRCSTDGCLVKKRVERDHEDPSYLVTTYEGIHNHTSPGIVYYAKQDSVSGRFHLSGS
>*Phoenix dactylifera*	FDISDYILSDEVTAPASFGQPESPVPPMVGVGQTATQTSSGSNLSAAGSSSSATRSAVERLRTDEGSKIAFRTKSEVEILDDGFKWRKYGKKSVKNSPNPRNYYRCSTEGCSVKKRVERDKEDPSYVITTYEGTHNHMSPGVVYYTTQDSTSGRFYVAGCELPPGS
>*Phragmites australis*	MAAVGARPALYHYQAPAGDALSMSSYFSHGGSSSSSSPSSFSAALGPAPLADPAAAQFDISEYLFDDAAAAVFAAPPADGGAPVVPDGAAAAAGAGAGATAHNARSAAEALPERPRTERIAFRTRSEIEILDDGYKWRKYGKKSVKNSPNPRNYYRCSTEGCNVKKRVERDKDDPSYVVTTYEGTHNHVSPSTVYYASHDAASGRFFVAGTQPPPGSLN
>*Rhynchospora breviuscula*	FDLSEYFIDEVSTHSSLLPPSYMDNSLFQAVQSSGVNLPVGGSGSVSKIAGMDKPRTERIAFRMKSVVEIIDDGFKWRKYGKKSVKNSPNPRNYYKCSTEGCSVKKRVEREKDDPSYVITTYEGIHNHVSPSMIYYTSHDAQSGQYYVSGYQISPGS
**>*Setaria italica***	**PRTTDRIAFRMRSEEEVLDDGYKWRKYGKKSVKNSPNPRNYYRCSTEGCNVKKRVERDKDDPSYVVTMYEGVHNHVSPGTIYYATQDAASGRFFVAGMHQFG**
**>*Sorghum bicolor***	**RTDRIAFRVRSDDEEVLDDGYKWRKYGKKSVKNSPNPRNYYRCSTEGCSVKKRVERDKDDQRYVVTMYEGVHNHVSPGTIYYATQDAASGRFFVAGMHQPG**
>*Stipagrostis hirtigluma*	MAAVGARPVLYHHPAPAAGDAYYSMSSYFSHGGSSTSSSASSFSAALGPAAPAPPPPIADPAAAQFDISEYLFDEGVFAAPPADAPPPVVAAPEGGSHGAGAGAVAHSARGAAAAAAEAVPERPRTERIAFRTRSEIEILDDGYKWRKYGKKSVKNSPNPRNYYRCSTEGCSVKKRVERDRDDPSYVVTTYEGTHNHVSPSTVYYASQDAASGRFFVAGTQPPGS
>*Triticum aestivum*	MAAVGAAPLLYQQQAQAVGDGCYFSSMSSQFSHGGISSTSSSPASSFSAALSATPAIAADPEAQFDISEYLFEEGAFSASLTPVVPVPAVAAAGASSATAVTARSAAESAAAAERPRTERIAFRTRSEIEILDDGYKWRKYGKKSVKNSPNPRNYYRCSTEGCNVKKRVERNRDDPAYVVTTYEGTHSHVSPSTVYYASQDAASGRFFVAGTHPPPGSLN

>*Triticum dicoccoides*	MAAVGAAPLLYQQHAQAAGDGYYFSFMSAYFSNGEASSNASSPASSFSAALGATPAI<br>AADPAAQFDISEYLFDEGAFSAALPPVVSVPAVGATAASSAIAVTARSAESAAAAERP<br>RTERIAFRTRSEVEILDDGYKWRKYGKKSVKNSPNPRNYYRCSTEGCNVKKRVERDR<br>DDPAYVVTTYEGTHSHVSPSTIYYASQDAASGRFFVAGTHPPPGSL
>*Triticum aestivum*	RPRTERIAFRTRTEIEILDDGYKWRKYGKKSVKNSPNPRNYYRCSTEGCSVKKRVERD<br>RDDPAYVVTTYEGTHSHVSPSTVYYASQDAASGRFFVAGTHPPPGSLN
>*Triticum urartu*	GAPLPVEAAVVPDVGYCDHTRAAAVAASGKIAFRTRSEEEILEDGYKWRKYGKKSV<br>KNSPNPRNYYRCSTEGCSVKKRVERDKDDANYVVTMYEGVHNHASPGTIYYAAQD<br>PASGRFFVTGTH
>*Typha angustifolia*	FDLSDFNLFDDGLALPQASFGQPVDSASTAPPLMESSGSGEMETPLTGRIAFRMKSEV<br>EILDDGFKWRKYGKKSVKNSPNPRNYYRCSTEGCSVKKRVERDREDPSYVVTTYEGV<br>HNHMSPGVVYYTTQDATSGQYLVAGCK
>*Urochloa decumbens*	MAAVGARPVLYHQYHHTAPAPAGDDAAAAGSMSSYFSHGGSSTSSSASASSFSAAL<br>GAAAVPAPPPLADQFDISEFLFDDGAGGVGGGAAQGVFAAAAPDGGPPPPPHAVP<br>AAGSAISAAAAHAARGAADQAMPERPRTERIAFRTRSEIEILDDGYKWRKYGKKSV<br>KNSPNPRNYYRCSTEGCNVKKRVERDKDDPSYVVTTYEGTHNHVSPSTVYYASQDA<br>ASGRFFVAGTQPPGSIN
>*Zea mays*	MAAVGAHPVLYHHPAPAGDASSMSSYFSHGGSSTTSSSASSFTAALAPTTTALAEHF<br>DISEFLFDDAAGAGVAGAPGVFADGAARPVVLPVPDAAGGGAIIGAAAGGAAAAS<br>EVPERPRTTRIAFRTRSEIEILDDGYKWRKYGKKSVKNSPNPRNYYRCSTEGCNVKKR<br>VERDKDDPSYVVTTYEGMHNHVSPSTVYYASQDAASGRFFVAGTQPPGSLN
>*Zingiber officinale*	TSRIAFRMKSVAEVLDDGFKWRKYGKKSVKNSPNPRNYYRCSTEGCSVKKRVERDK<br>DDPSYVITTYDGAHNHMSPGVVYYTTQDSVSGRFYVAGVQ
**>*Zizania palustris***	**MAAVGAHAVLYQCHHPVSAPASDAYSMSSYFSHRGSSTSSSASSFSAAALGATP<br>TSPLADPVAAQFDISEFFFDDTPAQAAAFPTPNGQPAVLPDGAAAASATAHARS<br>AAEAVPAPPVERPRTGRIAFRTRSEIEILDDGYKWRKYGKKSVKNSPNPRNYYRC<br>STEGCNVKKRVERDKDDPSYVVTTYEGTHNHVSPSTVYYASQDAASGRFFVAG<br>AQPPAGSLN**
>*Zizania latifolia*	IFDYLSDDQVRPPATVPGAFVAPSAMDPAEPAVVPDAAAGCYPPPRTMAAAMAGE<br>GRARITTDRIAFRTRSDDEILDDGYKWRKYGKKSVKNSPNPRNYYRCSTEGCNVKKR<br>VERDKNDPSYVVTTYEGIHNHVSPGTVYYAAQDAASGRFFVAGMHHPELN

Bolded sequences correspond to those presented in Figure 2A, and are formatted in bold for ease of reference.

Table S3 Cis-acting elements in the *OsWRKY7* promoter region

name	sequence	number	function
ARE	AAACCA	1	cis-acting regulatory element essential for the anaerobic induction
TCT-motif	TCTTAC	1	part of a light responsive element
CAT-box	GCCACT	1	cis-acting regulatory element related to meristem expression
TATA-box	TATATA	198	core promoter element around -30 of transcription start
G-box	CACGTC	3	cis-acting regulatory element involved in light responsiveness
TCCC-motif	TCTCCCT	1	part of a light responsive element
TGACG-motif	TGACG	2	cis-acting regulatory element involved in the MeJA-responsiveness
I-box	gGATAAGGTG	1	part of a light responsive element
Box 4	ATTAAT	1	part of a conserved DNA module involved in light responsiveness
GATA-motif	GATAGGG	2	part of a light responsive element
RY-element	CATGCATG	1	cis-acting regulatory element involved in seed-specific regulation
CAAT-box	CAAAT	9	common cis-acting element in promoter and enhancer regions
MRE	AACCTAA	3	MYB binding site involved in light responsiveness
GA-motif	ATAGATAA	1	part of a light responsive element
ABRE	ACGTG	3	cis-acting element involved in the abscisic acid responsiveness
3-AF1 binding site	TAAGAGAGGAA	1	light responsive element
GT1-motif	GGTTAA	3	light responsive element
CGTCA-motif	CGTCA	2	cis-acting regulatory element involved in the MeJA-responsiveness
GC-motif	CCCCCG	1	enhancer-like element involved in anoxic specific inducibility
O2-site	GATGATGTGG	1	cis-acting regulatory element involved in zein metabolism regulation
AuxRR-core	GGTCCAT	1	cis-acting regulatory element involved in auxin responsiveness

Table S4 Haplotype analysis of OsWRKY7

	Name	Position	ObsHET	PredHET	HWval	%Geno	FamTrio	MendErr	MAF	Alleles
1	26682761	26682761	0.002	0.027	4.0539E-82	99.7	0	0	0.014	C:A
2	26682916	26682916	0	0	1	99.4	0	0	0	G:T
3	26682978	26682978	0.019	0.486	0	97.7	0	0	0.415	A:C
4	26683002	26683002	0	0	1	97.7	0	0	0	A:C
5	26683015	26683015	0	0	1	99.7	0	0	0	C:T
6	26683179	26683179	0.001	0.002	0.0051	97.9	0	0	0.001	G:C
7	26683194	26683194	0	0.003	1.6982E-13	98.2	0	0	0.001	G:A
8	26683200	26683200	0	0	1	98.1	0	0	0	C:A
9	26683231	26683231	0	0	1	99	0	0	0.001	A:G
10	26683269	26683269	0.002	0.001	1	99.2	0	0	0.001	C:T
11	26683642	26683642	0	0.002	0.0003	99.7	0	0	0	C:T
12	26683696	26683696	0	0	1	99.6	0	0	0	G:C
